# Diversity and vertical distribution patterns of wildlife in Tianzhushan, Shaanxi Province, China

**DOI:** 10.3897/BDJ.10.e79923

**Published:** 2022-02-07

**Authors:** Yuting Sun, Han Hu, Congran Gong, Dongdong Yang, Lina Su, Peiwei Li, Yinhu Li, Yan Liu, Xiaomin Wu, Hongfeng Zhang

**Affiliations:** 1 Shaanxi Institute of Zoology, Xi’an, China Shaanxi Institute of Zoology Xi’an China

**Keywords:** Qinling Mountains, infrared camera, ungulates, relative abundance index (RAI), activity patterns, elevational distribution, seasonal variation

## Abstract

Biodiversity monitoring is an important means by which to evaluate management effectiveness and develop sound conservation plans. In this study, 52 cameras were installed in the study area of Tianzhushan to assess wildlife diversity and elevational patterns from 2018 to 2019. In total, 9 541 independent photos were collected within 26 565 camera-days. We analysed the relative abundance index (RAI), activity patterns and seasonal activity rhythms of the five most abundant species at 200-m elevational intervals throughout the study area (elevation range 1 400–2 000 m a.s.l.). Based on RAI results, the activity patterns of the five most abundant species (i.e. *Susscrofa*, *Naemorhedusgriseus*, *Muntiacusreevesi*, *Arctonyxcollaris* and *Capricornismilneedwardsii*) were analysed. Amongst the detected wild mammals, *Susscrofa* had the highest RAI value of 9.91, while the occurrence of *Tamiopsswinhoei* had the lowest RAI at 0.004. In addition, there were significant differences in species activity during the daytime and night-time. RAI of the ungulate community peaked at the 1 600–1 800 m a.s.l. elevational range, thus showing a mid-elevational peak pattern. Differences in vertical distribution patterns were detected for the five most abundant species. Mainland serows and wild boars preferred mid-elevation habitats (1 600–1 800 m a.s.l.), long-tailed gorals preferred mid- and high altitudes (1 600–2 000 m a.s.l.) elevation habitats and Chinese muntjacs and hog badgers preferred low elevation habitats (1 400–1 600 m a.s.l.). Taken together, this is the first study to reveal the five dominant species activity patterns in Tianzhushan, which is of importance for wildlife conservation.

## Introduction

Biodiversity represents the richness of life forms in a certain region and includes ecosystem, genetic and species diversity. Species diversity reflects the complex relationships amongst organisms, biological resources and the environment. Wild animals are an important link between the energy flow and material circulation within an ecosystem (Jiang 2001) and are involved in the maintenance of normal ecosystem function. Due to their high diversity, wide distribution and sensitivity to changes in habitat, mammals are considered as a key indicator of biodiversity conservation and environmental assessment. Over the last several decades, rapid economic development, expanding urbanisation and increasing transportation networks have led to severe fragmentation of natural habitats, as well as illegal poaching of threatened mammals. In the face of declining mammal populations, it is particularly important to carry out animal monitoring to clarify living status, population dynamic changes and threatened status and to propose targeted protection measures.

Compared with traditional survey methods, camera-trapping techniques have obvious advantages in their ability to monitor and study large and medium-sized mammals. Camera traps provide continuous monitoring and real imaging of animals within potentially difficult habitats and, thus, can be used to evaluate animal resources and monitor endangered species with minimal disturbance. In recent years, an increasing number of studies have used infrared camera technology to explore animal species diversity and wildlife activity. For example, Azlan and Sharma (2006) used camera traps to explore the diversity and activity patterns of wild felids in the Jerangau Forest Reserve in Malaysia. Liu et al. (2013) analysed mammal diversity and activity rhythms in different habitats in Guanyin Mountain Nature Reserve in the Qinling Mountains of China. Peng et al. (2021) used infrared cameras to investigate the activity rhythms of artiodactyls in the Baishuihe National Nature Reserve in China. Mori et al. (2020) investigated the activity rhythms of invasive coypus (*Myocastorcoypus*) using camera trapping. Scientists have also explored the vertical distribution of mammals using infrared cameras (Yan et al. 2019, Li et al. 2020). This technology has become a routine monitoring method for large- and medium-sized terrestrial mammals and birds (Rovero et al. 2010, Bridges and Noss 2011).

The Qinling Mountains not only form a dividing line between the Yangtze and Yellow River watersheds, but are also a transition zone between subtropical and warm-temperate climate zones. As such, they possess a rich and unique biodiversity and are of considerable research value. Tianzhushan Nature Reserve, which was initially established for the conservation of the forest musk deer (*Moschusberezovskii*) and its habitat, harbours a high species diversity. However, little is known about the status of mammalian biodiversity in this area of the Qinling Mountains. Thus, in the current study, we aimed to identify the dominant species in the study area, analyse their activity patterns and assess their elevational patterns. The results obtained in this study will provide important information for ecological research and support for the development of conservation measures for the dominant species in the area.

## Materials and methods

**Research area**

Tianzhushan Nature Reserve is located on the southern slopes of the Qinling Mountains (109°10′~110°03′E, 33°20′~33°23′N). Established in 2001, the Rreserve covers a total area of 21 685 hectares, including a core area of 7 541 hectares. The reserve has a temperate monsoon sub-humid mountain climate, with an altitudinal range of 800–2 074 m a.s.l., average annual temperature of 10–13°C, average annual rainfall of > 700 mm and frost-free period of 200 days. The area contains three major forest types: i.e. conifer forest, mixed conifer-broadleaf forest and deciduous broadleaf forest.

**Camera trapping**

**Figure 1.**
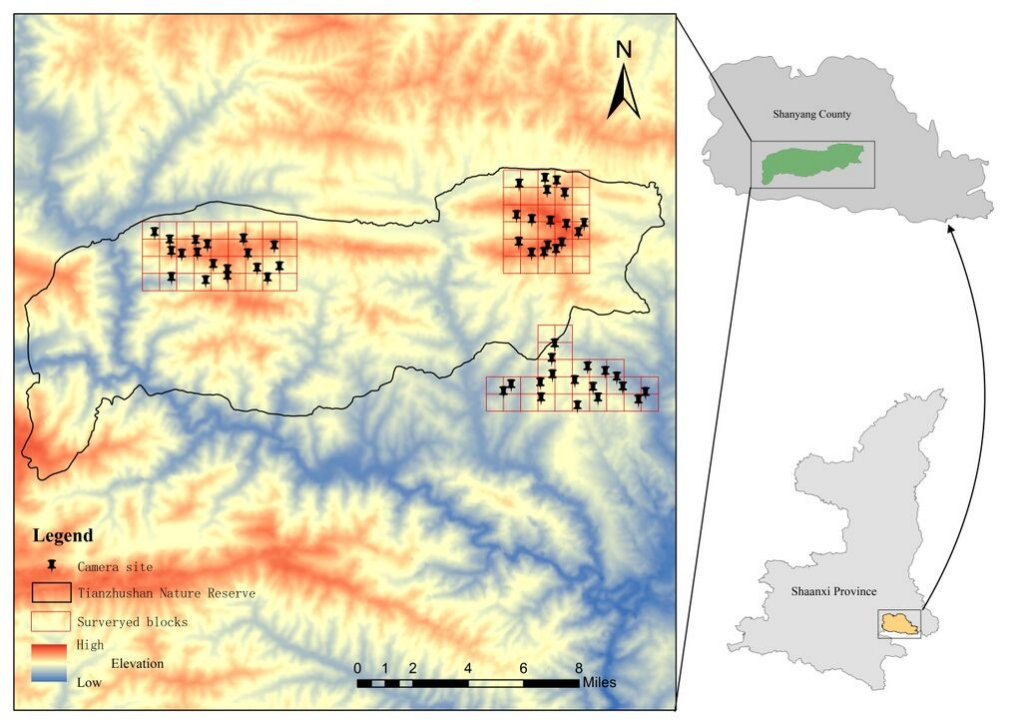
Camera trapping sites in Tianzhushan, Shaanxi Province, China.

Three observation sites, suitable for large- and medium-sized mammals to hunt or forage for food, were selected. Two sites were located within the reserve and one was located outside the reserve (elevation 1 400–2 000 m a.s.l.). Each site covered an area of 20 km^2^, with a distance of at least 3 km between sites. Each observation site was divided into 1 × 1 km^2^ grids (20 grids for each sample site). The distance between two camera traps in the different grids was at least 500 m (Fig. 1).

In total, 52 cameras were fixed on trees about 60 cm above the ground. The camera sensor was parallel to the ground to avoid direct sunlight. Cameras were configured to capture events with 15 s and infrared sensor sensitivity was configured to ‘medium’. Camera trapping data were downloaded every 3 months.

After the image data were collected, Bio-Photo v.2.1 was used to extract basic photo and video information. Excel data forms were exported for sorting and analysis. Images were classified and species were identified according to mammals, birds, livestock, poultry, staff and non-staff. Photos of the same species taken by the same camera after a 30-min interval were classified as an effective independent photo (Kawanishi et al. 1999, O'Brien et al. 2003). One camera working 24 h was regarded as a camera-day. We analysed two years of data collected between January 2018 and December 2019. In total, 5 991 independent photos of ungulates were collected over 26 565 camera-days.

**Data analysis**

**Relative abundance analysis**

The relative abundance index (RAI) is a standardised metric of how frequently a species appears on a camera (Botts et al. 2020). We calculated the RAI based on the following formula of O'Brien et al. (2003) and Botts et al. (2020):

RAI = A_i_/N × 100 (1)

where A_i_ represents the total independent photos of a species and N is the total number of camera-days. We calculated RAI separately for the first (RAI-2018) and second years (RAI-2019).

**Activity pattern analysis**

Based on RAI, the activity patterns of the five most abundant species were analysed. Monthly RAI (MRAI) and seasonal RAI (SRAI) were used to analyse monthly and quarterly activity rhythms, respectively:

MRAI = M_i_/T_i_ × 100 (2)

SRAI = N_j_/T_j_ × 100 (3)

where M_i_ is the total number of independent photos of each of the five species detected each month (i = 1…12); N_i_ is the total number of independent photos of each of the five species detected each season (j = spring, summer, autumn and winter); T_i_ is the number of camera-days each month; and T_j_ is the number of camera-days each season.

The daily activity patterns of the five most abundant species were analysed using time-period RAI (TRAI), defined following Liu et al. (2013):

TRAI = T_ij_/N_i_ × 100 (i = 1–5; j = 1–12) (4)

where the whole day (24 h) is divided into 2 h periods; T_ij_ is the number of independent photos of a species in one of 12 time periods (i = 1–5; j =1–12); and N_i_ is the total number of independent photos of a species over all time periods.

**Vertical distribution pattern analysis**

The vertical distribution patterns of the five most dominant species were also investigated. The study area (elevation range 1 400–2 000 m a.s.l.) was divided into three altitude ranges with vertical intervals of 200 m. The RAI of the target species within each altitude range was calculated using equation (1), where A_i_ is the independent photos of a species detected at one of three altitudes and N is the number of camera-days at one of three altitudes.

We next analysed the SRAI of each species detected at each altitude range, where N_ij_ is the number of independent photos of a species (i = 1…5) at one of three altitudes in each season (j = spring, summer, autumn and winter); and T_j_ is the number of camera-days at one of three altitudes in each season.

## Results

**Relative abundance of mammals**

**Figure 2.**
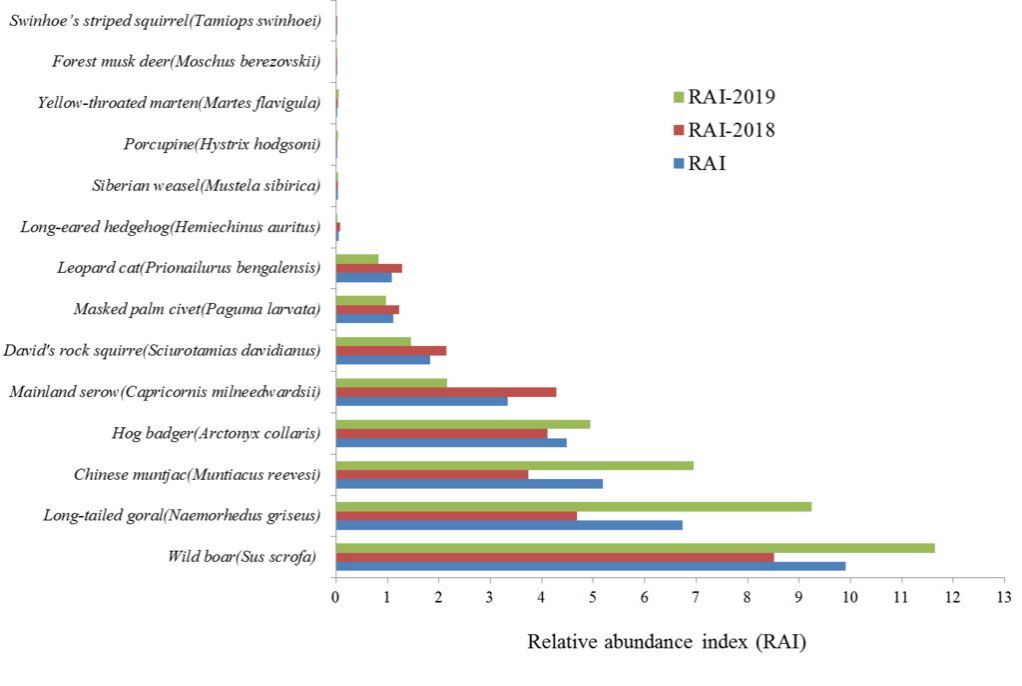
Relative abundance index (RAI) of ungulate species detected at infrared camera trap sites (RAI: Entire study period; RAI-2018: January–December 2018; RAI-2019: January–December 2019).

Between January 2018 and December 2019, 14 species of mammals were detected at the 52 infrared camera sites. Species included the forest musk deer, which is a Class I National Protected Species in China and listed as endangered on the IUCN Red List, as well as the long-tailed goral (*Naemorhedusgriseus*), mainland serow (*Capricornismilneedwardsii*) and yellow-throated marten (*Martesflavigula*), which are listed as Class II National Protected Species. The wild boar was detected most often (RAI = 9.91), followed by the long-tailed goral (RAI = 6.73), Chinese muntjac (*Muntiacusreevesi*, RAI = 5.18), hog badger (*Arctonyxcollaris*, RAI = 4.49) and mainland serow (RAI = 3.33), with the forest musk deer (RAI = 0.01) and swinhoe’s striped squirrel (*Tamiopsswinhoei*, RAI = 0.004) found at the lowest rates. RAI values for the first (RAI-2018) and second year (RAI-2019) differed somewhat (Fig. 2), suggesting temporal changes in the relative abundance of various species.

**Daily activity patterns of five most abundant species**

**Figure 3.**
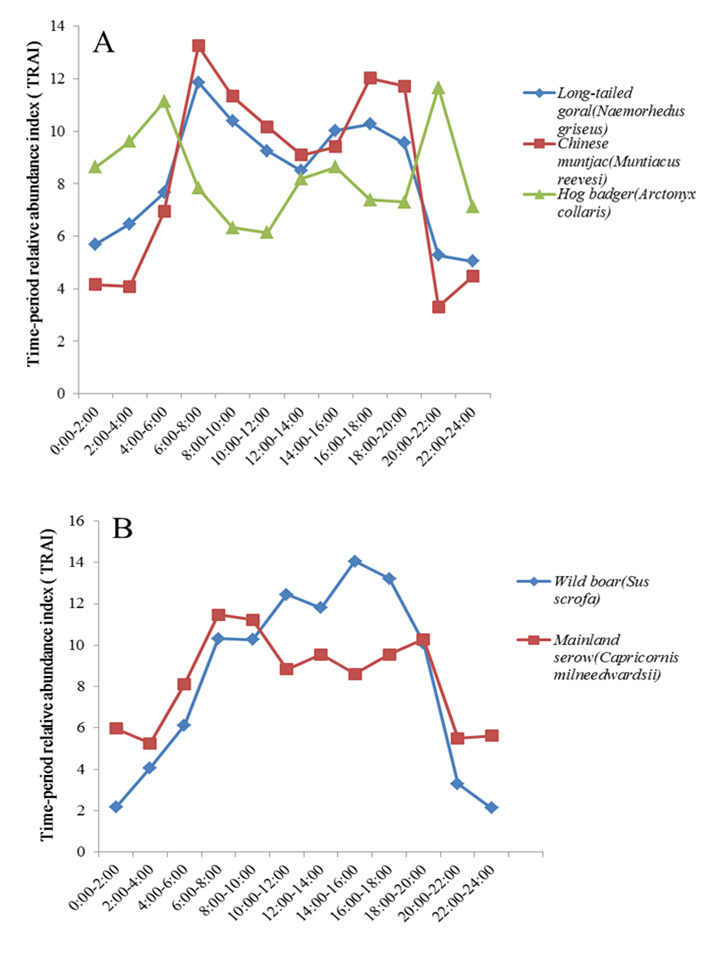
Daily activity patterns of five most abundant species at Tianzhushan. **A** Long-tailed goral, Chinese muntjac and Hog badger; **B** Wild boar and Mainland serow.

The daily activity patterns of the five most abundant species in Tianzhushan were analysed, based on TRAI (Fig. 3). Results showed that daily activity of the long-tailed gorals peaked at 06:00–08:00 and remained active continuously from 09:00 to 20:00 after the morning peak. The Chinese muntjacs were more active between 06:00 and 08:00 and between 16:00 and 20:00. Wild boars and mainland serows were more active from 06:00 to 20:00 and the highest activity period for wild boars appeared from 14:00 to 16:00. Hog badgers showed the opposite pattern, with lower activity during the day (lowest period between 10:00 and 12:00) and higher activity at night, peaking at 20:00–22:00 and 04:00–06:00.

**Annual activity patterns of five most abundant species**

**Figure 4.**
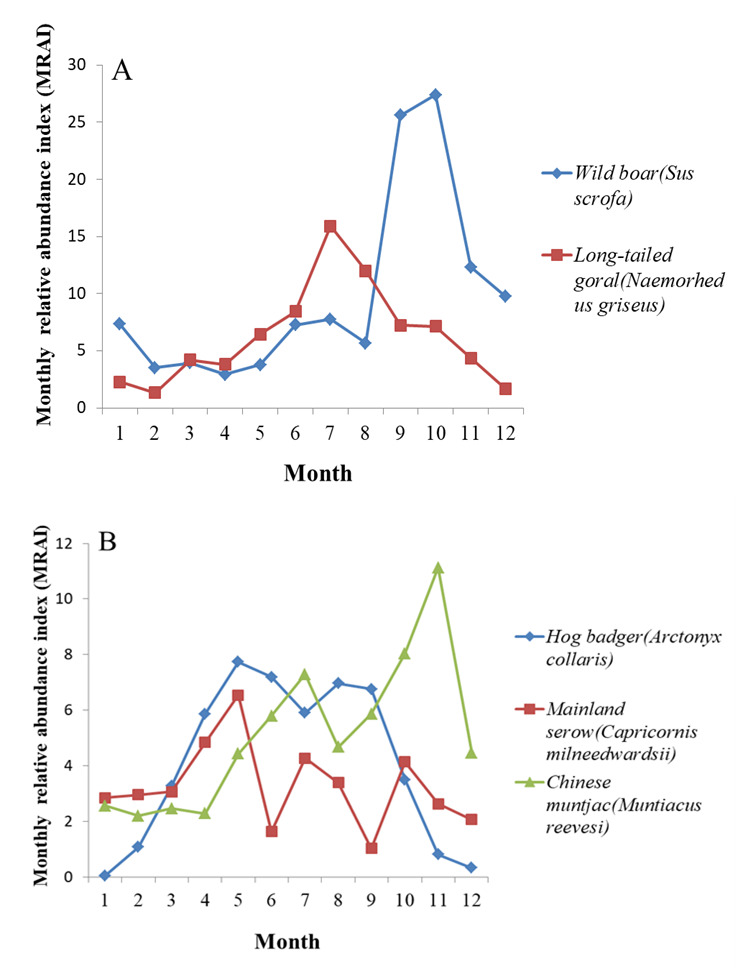
Annual activity patterns of five most abundant species at Tianzhushan. **A** Long-tailed goral and wild boar; **B** Hog badger, Chinese muntjac and Mainland serow.

The annual activity patterns of the five most abundant species are shown in Fig. 4. Results showed that wild boars were more active from September to November, with a peak in October. Long-tailed gorals were most active in July, with low activity from November to April (winter season). Chinese muntjacs showed activity peaks in July and November. Hog badgers showed two activity peaks in May and August. Mainland serows displayed a different pattern, with activity peaks in May, July and October.

**Vertical distribution patterns of five most abundant species**

**Figure 5.**
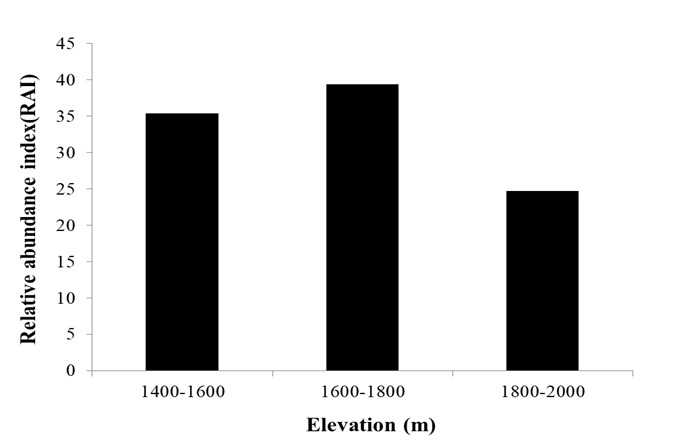
RAI of ungulates at Tianzhushan along the elevational gradient.

**Figure 6.**
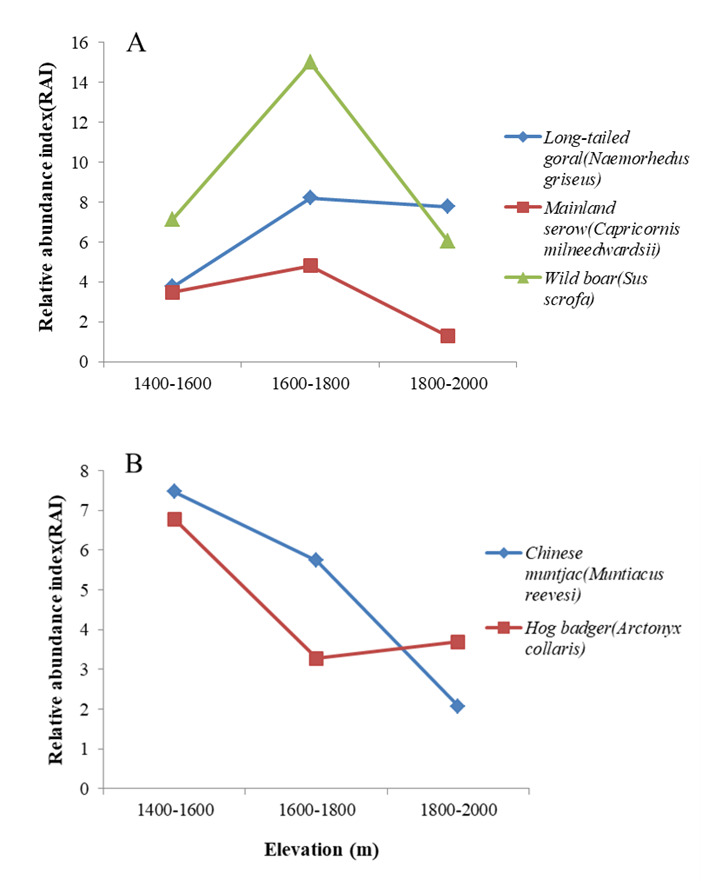
RAI of five most abundant species at each elevational gradient at Tianzhushan. **A** Long-tailed goral, Mainland serow and Wild boar; **B** Hog badger and Chinese muntjac.

The RAI values of forest ungulates were calculated at 200 m elevational intervals throughout the study area (elevation range 1 400–2 000 m a.s.l.). Results showed that the relative abundance of forest ungulates was higher within the 1 600–1 800 m a.s.l. range than at other elevations (Fig. 5). We next calculated the RAI of the five most abundant species at each elevational gradient (Fig. 6). All five species were found at all elevational gradients. Mainland serows and wild boars preferred mid-elevation habitats (1 600–1 800 m a.s.l.). For long-tailed gorals, RAI was higher at mid- and high altitudes (1 600–2 000 m a.s.l.) and lower at low altitude. For Chinese muntjacs, RAI decreased with elevation, with a peak at 1 400–1 600 m a.s.l. For hog badgers, the RAI was lowest at mid-altitude (1 600–1 800 m a.s.l.).

**Vertical distribution patterns of five most abundant species in Tianzhushan by season**

**Figure 7.**
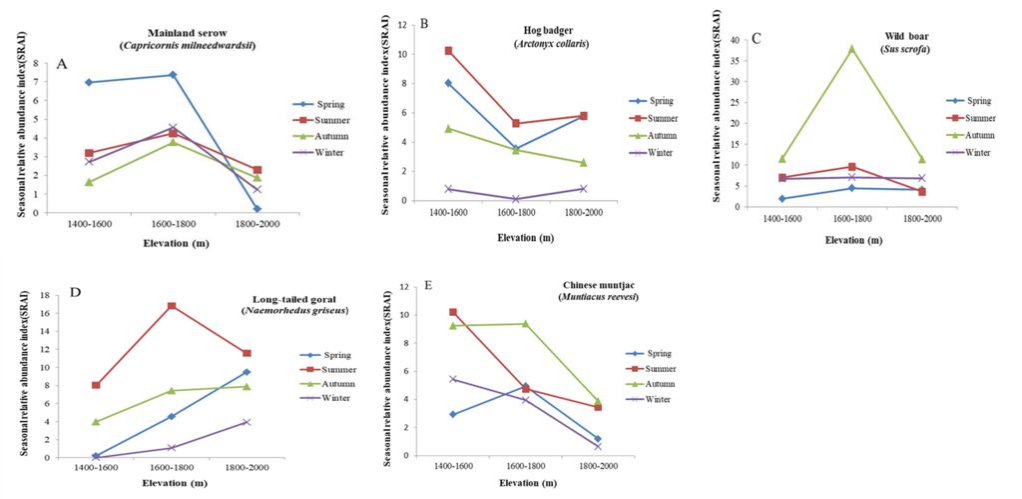
Vertical distribution patterns of five most abundant species by season at Tianzhushan. **A** Mainland serow; **B** Hog badger; **C** Wild boar; **D** Long-tailed goral; **E** Chinese muntjac.

We performed comparative analyses of vertical distribution patterns of the dominant species by season (Fig. 7). Mainland serows showed similar vertical distribution patterns in all four seasons, with the highest RAI at the mid-altitude range (1 600–1 800 m a.s.l.). Wild boars showed a consistent vertical distribution pattern in all four seasons, with the highest population found at the 1 600–1 800 m a.s.l. elevation range and autumn recorded much higher RAI for wild boars. Hog badgers showed a similar vertical distribution pattern in spring, summer and winter and the populations of hog badger inhabiting the altitude of 1 600 to 1 800 m a.s.l. is lower than those in other altitudes, but in autumn, it is lower at 1 800–2 000 m a.s.l.. Long-tailed goral populations were highest at 1 800–2 000 m a.s.l. in autumn, spring and winter and lowest at 1 400–1 600 m a.s.l.. In contrast, the summer long-tailed goral population was highest at 1 600–1 800 m a.s.l.. Chinese muntjacs showed a similar vertical distribution pattern in autumn and spring (preferring mid-elevation habitats at 1 600–1 800 m a.s.l.) and a similar pattern in summer and winter (preferring low elevation habitats at 1 400–1 600 m a.s.l.).

## Discussion

This study is the first to monitor the Tianzhushan Nature Reserve in Shaanxi Province via infrared camera. In total, 14 mammal species were detected, including one endangered Class I National Protected Species and three Class II National Protected Species. The five species with the highest RAI were *Susscrofa*, *Naemorhedusgriseus*, *Muntiacusreevesi*, *Arctonyxcollaris* and *Capricornismilneedwardsii*. The RAI of the wild boars was significantly higher than that of the other species. Strong environmental adaptability, high reproduction rate and widespread lack of large predators in the reserve have likely contributed to the higher wild boar populations (Frauendorf et al. 2016, Johann et al. 2020). Although it is a key protected animal in the reserve, the RAI of the forest musk deers was relatively low, which may be related to the significant decline in wild forest musk deer populations due to habitat loss and overexploitation (Yang et al. 2003, Fan et al. 2018). Swinhoe’s striped squirrels had the lowest RAI value, which is likely due to the low record of small mammals in the study area and limited camera coverage. Another reason for the low record could be related to the fact that swinhoe’s striped squirrels are an arboreal species.

Animal activity intensity in different time periods is positively correlated with the probability of being captured by the cameras. The higher the activity intensity index, the more active animals are at this time (Azlan and Sharma 2006). According to the daily activity rhythms of the five most abundant species, Chinese muntjacs were more active from 06:00 to 08:00 and 16:00 to 20:00, indicating higher activity at dawn and dusk, consistent with reports in different areas of the Qinling Mountains (Jia et al. 2014). This daily activity pattern is essential for herbivores as morning foraging on dew-covered plants can provide additional water and evening activity can reduce sun exposure to avoid water loss. In addition, the low light at dawn and dusk can reduce the risk of predation (Yuan and Kong 2011). Our results also showed that long-tailed gorals were more active from 06:00 to 20:00, suggesting that they are a diurnal species, as reported in previous research (Jia et al. 2014). In our study area, wild boars were more active during the day than at night. This is similar to results reported in Guanyinshan Nature Reserve in Shaanxi, China (Liu et al. 2013) and in Saskatchewan, Canada (Stolle et al. 2015), but different from research in south-western Germany (Johann et al. 2020) and the Mediterranean (Russo et al. 1997), which reported predominantly nocturnal activity. The different results between regions in terms of daily activity patterns may be due to local environmental conditions. Our results also showed that mainland serows were predominantly active during the day, whereas hog badgers showed obvious nocturnal habits.

Analysis of the annual activity patterns showed that activity intensity for the five most abundant species fluctuated seasonally, which may be related to the different distribution of food resources (Chen and Hu 2012), as well as changes in temperature, light and water availability at different times of the year (Wang et al. 2008). In winter, the relative abundance and activity intensity of the five species were low, which coincided with the lowest levels of food availability, shortest sunshine hours and lowest temperatures. Wild boars were more active in September and October, corresponding to their breeding season when the frequency and range of activities increase in order to find a mate. Mainland serows, Chinese muntjacs and long-tailed gorals were highly active in the summer month of July, coinciding with the increase in food abundance and, thus, foraging activity. Mainland serows activity also peaked in May, which may be due to the increase in food resources in spring, especially the stems and buds of favoured plants. Mainland serows were also active in October, which may be related to their breeding behaviour. For hog badgers, it was already reported to have a period of winter torpor or (semi-) hibernation from November to March in central and northern China (Wang and Fuller 2003, Zhou et al. 2015) . This may explain the fact that hog badgers were more active from March to October.

The spatial patterns of species richness and diversity are of major interest in ecology and biogeography. Species diversity distribution is affected by many ecological gradients (Gaston 2000, Morin 2000), with highest species diversity found at mid-altitudes (Rickart 2010). Here, we calculated the RAI for all forest ungulates at different elevations and found that the spatial distribution of forest ungulates was highest at mid-elevation (1 600–1 800 m a.s.l.), thus showing a mid-peak model, in accordance with the prediction of the mid-domain effect hypothesis. This model is consistent with the results of Zeng et al. (2005) and Li et al. (2014) in different regions of the Qinling Mountains. Our results also showed that hog badgers preferred low elevation habitats (1 400–1 600 m a.s.l.), mainland serows and wild boars preferred mid-elevation habitats (1 600–1 800 m a.s.l.), long-tailed gorals preferred mid- and high-altitude areas (1 600–2 000 m a.s.l.) and Chinese muntjacs preferred low to mid-altitude habitats (1 400–1 800 m a.s.l.). Species preferring different elevation habitats may be a response to differences in habitat adaptability (SÁnchez-Cordero 2001).

Considering the seasonal vertical migration behaviour of ungulates (Herrero et al. 1996), we analysed the vertical distribution patterns of the five most abundant species in the different seasons. In the four seasons, there was no change in the vertical distribution pattern of mainland serows and wild boars, suggesting they had no obvious vertical migration behaviour. The spatial distribution of artiodactyls varies in different regions (Gonzalez 1985). For example, different from our results, mainland serows migrate downwards in spring and upwards in summer in the Cibaigou Nature Reserve in Tibet (Wu and Zhang 2004). It is possible that, due to the elevation range (1 580–5 000 m a.s.l.) being much higher there, the habitat heterogeneity is much more marked in Cibaigou Nature Reserve in Tibet than in our study area, which may contribute to this difference. However, the real reason still needs more study in the future. Zhou et al. (2015) and Kaneko et al. (2006) had reported strong seasonal variations in the diet composition of hog badgers in a Chinese subtropical forest and Japanese badgers in Hinode, Japan. They reported that, during spring and summer, earthworms were the predominant food category and badgers exhibited switched from worms when fruit became abundant in autumn. Thus, the vertical distribution of hog badgers in autumn was different to that in other seasons, which may be related to seasonal changes in food availability. The long-tailed gorals preferred mid-elevation habitats in summer, but migrated upwards in spring and winter. This may be due to animals travelling to higher altitudes in winter and spring to gain more solar radiation in cold environments (Zeng et al. 2010). The Chinese muntjacs were primarily distributed at mid-elevation in spring, at low elevation in summer and winter and at both during autumn. This seasonal variation in the vertical distribution could be attributed to the seasonal changes in food resources.

## Conclusions

In conclusion, our data revealed the survival status and activity rhythms of five most abundant species in Tianzhushan, thus providing a strong basis for the efficient monitoring, protection and management of ungulates. However, further research is needed to understand the dominant factors affecting ungulates activity rhythms and the relationship between ungulates and predators.

## Hosting institution

Shaanxi Institute of Zoology

## References

[B7611129] Azlan J. M., Sharma D. S.K. (2006). The diversity and activity patterns of wild felids in a secondary forest in Peninsular Malaysia. Oryx.

[B7611263] Botts R. T., Eppert A. A., Wiegman T. J. (2020). Circadian activity patterns of mammalian predators and prey in Costa Rica. Journal of Mammalogy.

[B7611229] Bridges A. S., Noss A. J., O’Connell A. F., Nichols J. D., Karanth K. U. (2011). Camera traps in animal ecology.

[B7611452] Chen W, Hu J. (2012). Seasonal differences in microhabitat use by tufted deer (*Elaphoduscephalophus*) in Tangjiahe Nature Reserve. Acta Theriologica Sinica.

[B7611353] Fan Z., Li W., Jin J. (2018). The draft genome sequence of forest musk deer (*Moschusberezovskii*). Oxford Open.

[B7611334] Frauendorf M., Gethöffer F., Siebert U. (2016). The influence of environmental and physiological factors on the litter size of wild boar (*Susscrofa*) in an agriculture dominated area in Germany. Science of the Total Environment.

[B7616409] Gaston K. J. (2000). Global patterns in biodiversity. Nature.

[B7633192] Gonzalez G., Lovari S. (1985). The biology and management of mountain ungulates.

[B7616487] Herrero J., Garin I., García-Serrano A. (1996). Habitat use in a *Rupicaprapyrenaicapyrenaica* forest population. Forest Ecology and Management.

[B7611101] Jiang Z. (2001). Values and ecological service functions of wildlife. Acta Ecologica Sinica.

[B7611362] Jia X., Liu X., Yang X. (2014). Seasonal activity patterns of ungulates in Qinling Mountains based on camera-trap data. Biodiversity Science.

[B7611324] Johann F., Handschuh M., Linderoth P. (2020). Adaptation of wild boar (*Susscrofa*) activity in a human-dominated landscape. BMC Ecology.

[B7656337] Kaneko Y., Maruyama N., Macdonald D. W. (2006). Food habits and habitat selection of suburban badgers (*Melesmeles*) in Japan. Journal of Zoology.

[B7611243] Kawanishi K., Sahak A. M., Sunquist M. (1999). Preliminary analysis on abundance of large mammals at Sungai Relau, Taman Negara. Journal of Wildlife and Parks.

[B7611196] Li J., Xue Y., Zhang Y. (2020). Spatial and temporal activity patterns of golden takin (*Budorcastaxicolorbedfordi*) recorded by camera trapping. PeerJ.

[B7616448] Li S., Zhang X., Chen P. (2014). The community structure and elevational patterns of forest ungulates at the southern slope of the Qinling Mountains, China. Chinese Journal of Zoology.

[B7611151] Liu X., Wu P., Songer M. (2013). Monitoring wildlife abundance and diversity with infra-red camera traps in Guanyinshan Nature Reserve of Shaanxi Province, China. Ecological Indicators.

[B7611169] Mori E., Andreoni A., Cecere F. (2020). Patterns of activity rhythms of invasive coypus
*Myocastorcoypus* inferred through camera-trapping. Mammalian Biology.

[B7616419] Morin P. J. (2000). Biodiversity's ups and downs. Nature.

[B7611253] O'Brien T. G., Kinnaird M. F., Wibisono H. T. (2003). Crouching tigers, hidden prey: Sumatran tiger and prey populations in a tropical forest landscape. Animal Conservation.

[B7611160] Peng K., Chen X., Wen P. (2021). The activity rhythm survey of ungulates in Baishuihe National Nature Reserve based on infrared camera trapping. Journal of Sichuan Forestry Science and Technology.

[B7616429] Rickart E. A. (2010). Elevational diversity gradients, biogeography and the structure of montane mammal communities in the intermountain region of North America. Global Ecology and Biogeography.

[B7633525] Rovero F., Tobler M., Sanderson J., Eymann J., Degreef J., Hauser C. (2010). Manual on field recording techniques and protocols for all taxa biodiversity inventories and monitoring.

[B7611381] Russo L., Massei G., Genov P. V. (1997). Daily home range and activity of wild boar in a Mediterranean area free from hunting. Ethology Ecology and Evolution.

[B7616457] SÁnchez-Cordero V. (2001). Elevation gradients of diversity for rodents and bats in Oaxaca, Mexico. Global Ecology and Biogeography.

[B7611391] Stolle K., Van Beest F. M., Vander Wal E. (2015). Diurnal and nocturnal activity patterns of invasive wild boar (*Susscrofa*) in Saskatchewan, Canada. Canadian Field Naturalist.

[B7616399] Wang B., Zhang J., Hu J. (2008). Habitat selection by Chinese goral (*Naemorhedusgriseus*) in spring in Fentongzhai Nature Reserve. Sichuan Journal of Zoology.

[B7654482] Wang H., Fuller T. K. (2003). Food habits of four sympatric carnivores in southeastern China. Mammalia.

[B7616507] Wu P., Zhang E. (2004). Habitat selection and its seasonal change of serow (*Capricornissumatraensis*) in Cibagou Nature Reserve,Tibet. Acta Theriologica Sinica.

[B7611344] Yang Q., Meng X., Lin X. (2003). Conservation status and causes of decline of musk deer (*Moschus* spp.) in China. Biological Conservation.

[B7611187] Yan W., Ji S., Shuai L. (2019). Spatial distribution patterns of mammal diversity in Yangxian County of Shaanxi Province on the southern slope of the Qinling Mountains. Biodiversity Science.

[B7611371] Yuan B., Kong F. (2011). Research on mammal activity rhythm. Journal of Anhui Agricultural Sciences.

[B7616439] Zeng Z., Song Y., Ma Y. (2005). Fauna characteristics and ecological distribution of Carnivora and Artiodactyla in Niubeiliang Nature Reserve. Acta Ecologica Sinica.

[B7616517] Zeng Z., Beck P. S.A., Wang T (2010). Plant phenology and solar radiation drive seasonal movement of golden takin in the Qinling Mountains, China. Journal of Mammalogy.

[B7654471] Zhou Y., Chen W., Kaneko Y. (2015). Seasonal dietary shifts and food resource exploitation by the hog badger (*Arctonyxcollaris*) in a Chinese subtropical forest. European Journal of Wildlife Research.

